# Inhibition of Ice Growth and Recrystallization by Zirconium Acetate and Zirconium Acetate Hydroxide

**DOI:** 10.1371/journal.pone.0059540

**Published:** 2013-03-21

**Authors:** Ortal Mizrahy, Maya Bar-Dolev, Shlomit Guy, Ido Braslavsky

**Affiliations:** The Institute of Biochemistry, Food Science, and Nutrition, The Robert H. Smith Faculty of Agriculture, Food and Environment, The Hebrew University of Jerusalem, Rehovot, Israel; University of California at Berkeley, United States of America

## Abstract

The control over ice crystal growth, melting, and shaping is important in a variety of fields, including cell and food preservation and ice templating for the production of composite materials. Control over ice growth remains a challenge in industry, and the demand for new cryoprotectants is high. Naturally occurring cryoprotectants, such as antifreeze proteins (AFPs), present one solution for modulating ice crystal growth; however, the production of AFPs is expensive and inefficient. These obstacles can be overcome by identifying synthetic substitutes with similar AFP properties. Zirconium acetate (ZRA) was recently found to induce the formation of hexagonal cavities in materials prepared by ice templating. Here, we continue this line of study and examine the effects of ZRA and a related compound, zirconium acetate hydroxide (ZRAH), on ice growth, shaping, and recrystallization. We found that the growth rate of ice crystals was significantly reduced in the presence of ZRA and ZRAH, and that solutions containing these compounds display a small degree of thermal hysteresis, depending on the solution pH. The compounds were found to inhibit recrystallization in a manner similar to that observed in the presence of AFPs. The favorable properties of ZRA and ZRAH suggest tremendous potential utility in industrial applications.

## Introduction

Ice growth and nucleation play a significant role in life on earth, for example, in the freezing of environmental water resources, as well as the freezing of water in plant and animal tissue, both of which can result in fatal damage. Organisms living in cold environments must respond appropriately to survive when temperatures drop below the freezing point. Such organisms have evolved a series of mechanisms for controlling ice growth and protecting themselves from freezing damage, either by avoiding freezing or by tolerating the freezing process. Control over ice growth is crucial in industrial settings as well. Processes such as ice templating are used in the engineering of porous materials [1], [2]. In cryopreservation, control over ice growth is crucial to maintaining the viability of cells, tissues, and organs [3], [4].

Ice growth may be controlled by inhibiting ice recrystallization, in which the growth of large ice crystals is favored at the expense of small ones (Otswald ripening [5]). Ice recrystallization is lethal in tissues, for example in crops at times of a frost. This process produces mechanical damage to cell membranes during the freeze–thaw cycle of cryopreservation [3], [4], [6]. In the frozen food industry, ice recrystallization can avert food texture [7]. Substances that can inhibit ice recrystallization and control ice growth are, therefore, attractive for industrial and medical applications.

Ice binding proteins (IBPs) are a set of natural proteins that may be used to control ice growth and recrystallization. This expanding group of proteins interacts with ice for various biological purposes such as freeze avoidance, ice recrystallization inhibition (IRI), and ice nucleation promotion, thereby protecting organisms from ice damage [8], [9]. A subset of this group is the antifreeze proteins (AFPs), which were found to control ice shape, growth and melting in a non-colligative manner. AFPs can depress the freezing temperature of seed ice crystals below the melting point, resulting in a thermal hysteresis gap. AFPs were also shown to have IRI activities. AFPs are expressed in a variety of organisms that require survival in cold environments, including insects, fish, bacteria, plants, fungi, and plankton (reviewed in [8]). These proteins are excellent candidates for use in cryopreservation, the frozen food industry, and other fields in which control over ice growth and recrystallization are important. Although AFPs are currently used in industry, for example, in ice cream [10], the difficulties associated with producing AFPs in large quantities preclude them from use in other industrial applications. Furthermore, the lifetimes of AFPs are limited compared to other cryoagents. An ideal synthetic alternative cryoprotectant would be low in cost, readily available, and simple to use.

Non-protein cryoprotective agents (CPAs) that act in a colligative manner such as glycerol, dimethyl sulfoxide (DMSO), and certain sugars are widely used in cryopreservation. These materials can protect cells from damage caused by the formation of intracellular ice during freezing and thawing. CPAs require high concentrations to be effective and some display high cytotoxic effects [11]. Few additives have been identified as exhibiting non-colligative effects similar to those of AFPs upon crystal growth and recrystallization. These include block co-polymers based on poly(ethylene oxide) and polyethyleneimine modified with hydroxyl groups [12], polyvinyl alcohol (PVA) [13], synthetic peptides [14] and peptoids [15], and xylomannan, a substance containing mostly polysaccharides and fatty acids which was isolated from a freeze-tolerant beetle [16], [17]. Recently, the inorganic material zirconium(IV) acetate (ZRA) was shown to induce six-fold symmetry in cavities of materials prepared by ice templating over the pH range 3.5–4.5. These shapes are similar to ice shapes observed in the presence of a variety of AFPs [18]. Such shaping was not apparent in similar materials, such as yttrium acetate, barium acetate, or zirconium(IV) acetate hydroxide (ZRAH)] [19].

Here, we have expanded the study of ZRA and ZRAH by measuring their potential to act as cryoprotectants through direct ice shaping, modulating the growth rate of ice, and inhibiting ice recrystallization. ZRA and ZRAH induced hexagonal ice shaping, and both materials significantly reduced the rate of ice crystal growth compared to buffer, depending on the solution pH. ZRA and ZRAH were also shown to inhibit ice recrystallization in a manner similar to AFPs.

## Materials and Methods

### Solution Preparation

ZRAH powder and a ZRA solution were purchased from Sigma-Aldrich Ltd. ZRAH was dissolved in 100 mM acetate buffer to a final concentration of 30 g/L. This concentration provides 100–150 mM of Zr in the solution, as calculated using the empiric formula (CH_3_CO_2_)_x_Zr(OH)_y_, x+y∼4. The pH of the ZRAH solution was adjusted using 100 mM acetic acid for pH values of 3.3 and 4.2, and 100 mM sodium acetate for pH 4.7. The ZRA is provided by the manufacturer as 16% solution in dilute acetic acid with the formula Zr^x+^(CH_3_COOH)_x_. Using the given density of 1.279 g/ml, this solution contains 200 g/L or 2.2 M Zr. The solution was diluted either with pure water (for pH of 3.3) or with 100 mM sodium acetate (for pH values of 4.2 or 4.7) to obtain a final Zr concentration of 13.3 g/L (150 mM). The pH was adjusted using HCl. A sample of a recombinant type III AFP from Ocean pout was kindly provided by Peter L. Davies (Queen’s University) [20]. The AFP solution contained 40 µM protein in 100 mM ammonium bicarbonate at pH 8.

### Growth Rate Measurements

The ice growth rate was measured using a custom-designed nanoliter osmometer controlled by a LabView interface developed in our lab. The system and procedure are described in detail elsewhere [21],[22]. The temperature in this apparatus can be controlled with a precision of 2–3 millidegrees [22], [23]. Briefly, sub-microliter volumes of the solutions were injected into a temperature-controlled sample holder using a micropipette (prepared with a pipette puller device). The sample solution was flash frozen and the temperature was slowly increased toward the melting point until a small (10–20 µm diameter) single ice crystal remained. Once the single crystal was formed, its melting temperature (T_m_) was determined as the lowest temperature at which melting was observed. The temperature was then reduced to the desired supercooling temperature and held at this temperature while growth was recorded. The experiment was repeated at least three times on three different samples for each temperature.

Growth rates were determined from recorded images using an ImageJ analysis routine [24]. To determine the growth rate of each point, at least five snapshots were taken, one at the beginning of the cooling period and the others during the growth course. For each pair of snapshots, the distance between the crystal edges across the *c* and/or the *a* axis was measured and the difference between the measured distances divided by a factor of 2 was taken as the elongation of the crystal along the measured axis. The growth rate was determined as the division of the elongation by the time that passed during the growth course, and a mean value was calculated from the different snapshots for each experiment. The directionality of the ice crystals was determined according to the crystal symmetry (twofold symmetry in the *c* direction and six-fold symmetry in the *a*-direction). ZRA and ZRAH were tested for their effects on ice crystal growth over the pH range 3.3–4.7. pH 4.7 was the highest pH at which measurements could be collected. At higher pH values, the solutions polymerized and turned into gels. Even at pH 4.7, the solution became viscous and gelated after 4–6 days.

### Ice Recrystallization Inhibition (IRI) Activity

IRI experiments were performed using a Nikon optical microscope equipped with a LC-PolScope (Abrio IM™ Imaging System) and a Linkam MDBCS 196 cold stage (Linkam Scientific Instruments Ltd, UK). A sample of 5 µL was inserted into a 15 mm inner diameter quartz sample holder and covered with a circular glass coverslip. The quartz dish was then placed on a temperature-controlled silver block inside the cold stage. A flow of dry nitrogen was subjected on top to prevent humidity in the ambient air from condensing on the upper window. The experimental procedure was as follows: Samples were cooled from room temperature to –20°C at a cooling rate of 50°C/min. During this cooling phase, ice crystals spontaneously nucleated from the supercooled solution. Samples were kept at –20°C for 2 min, and during this time, images of the frozen samples were collected. The temperature was then warmed to –3°C at a constant rate of 5°C/min, and thereafter the temperature was maintained at –3°C for 10–60 min to permit recrystallization. During this time period the development of the ice crystals was recorded. With the LC-PolScope image system, multiple images with different polarization settings are recorded by a digital camera. These images were used to determine the optical axis in the sample fractions. A color gradation is used to indicate the orientations of the optical axis in each ice crystal. Three replicate experiments were performed, at a minimum, for each determination.

## Results

### Influence of ZRA and ZRAH on Ice Shaping

**Figure 1:**
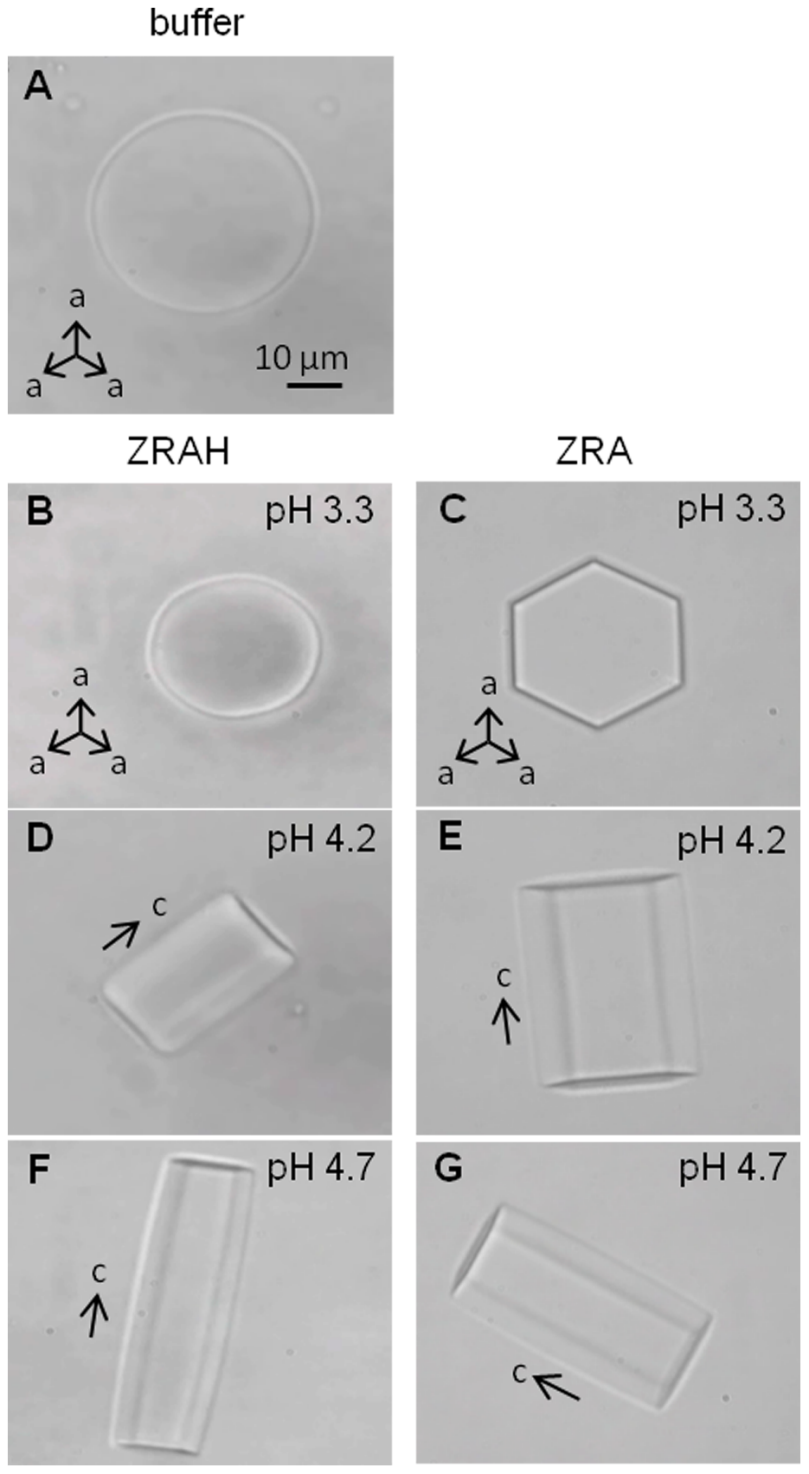
Ice crystal shaping during growth in the presence of (A) 100 mM sodium acetate (pH 4.2), (B, D, F) ZRAH (30 g/L) and (C, E, G) ZRA (13.3 g/L) solutions.

Ice in ZRAH solutions at pH 4.2 and 4.7 and ice in ZRA solutions at all pH values developed shapes that appeared to be faceted with hexagonal symmetry in the *a*-direction and twofold symmetry in the *c* direction (Fig. 1). These shapes were similar to those observed in the presence of several mutated AFPs [25], [26], AFP analogues [27], or AFPs at low concentrations [26], in accordance with the results of [18]. Crystals assumed the shapes of elongated hexagonal tubes and retained these shapes throughout the growth process. For ZRAH, some shrinking in the basal planes was occasionally detected during growth, leading to the development of truncated pyramidal shapes (Fig. S1). We did not detect the formation of full bipyramidal shapes in which basal planes are not exposed. Ice crystals grown in the ZRAH solution at pH 3.3 assumed flat disc shapes (Fig. 1b), similar to the shape observed in a buffer solution (Fig. 1a) or in pure water [28]. This circular shape resulted in the more rapid growth of the crystal in the directions perpendicular to the *c*-axis. Interestingly, at high supercooling levels, the crystals developed columnar hopper shapes with hollow ends (Fig. S2). Hopper crystal shapes have been described by Deville et al, albeit under different growth conditions [19]. In all cases, the faceting structures that were apparent during growth were lost upon melting, as has been observed in the presence of moderately active AFPs [29].

### Growth Rate Reduction and TH Activity

**Figure 2:**
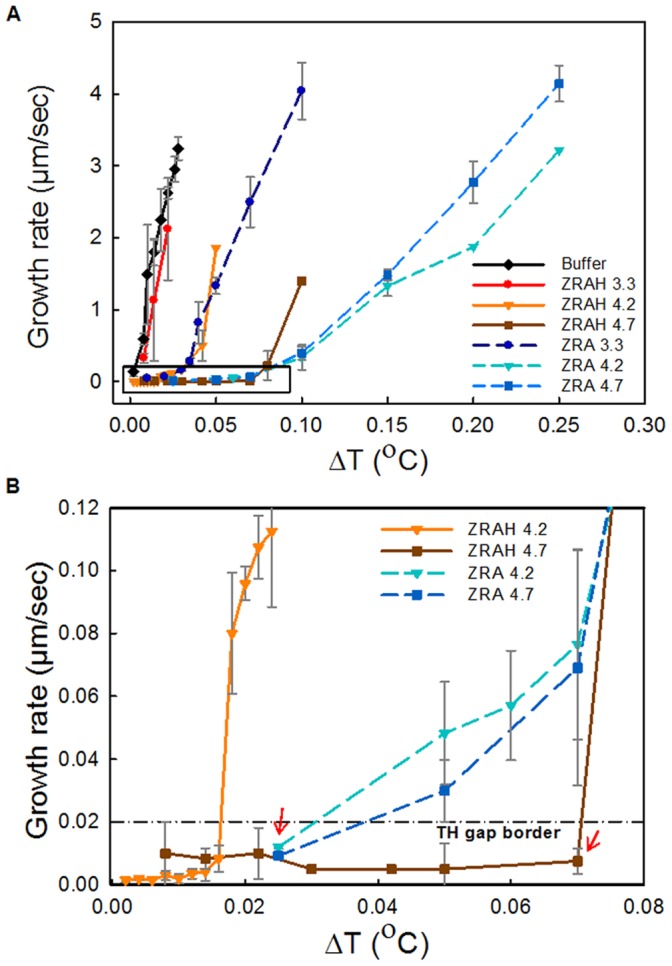
The effect of ZRA and ZRAH on the growth rate of ice crystals along the *a*-axis. The X-axis represents the temperature below T_m_. The Y-axis represents the rate of growth of the radius of the ice crystal. Each point represents the average of at least 3 measurements. (B) A magnified view of the area enclosed by the black rectangle in A. The horizontal dash-dot line indicates a growth rate of 0.02 µm/sec, which is the threshold value used to define the TH gap. The arrows indicate the semi-TH activity according to this modified definition. Note that according to the old threshold definition (0.2 µm/sec) the TH activity in the *a*-axis direction would be >0. 1°C.

**Figure 3:**
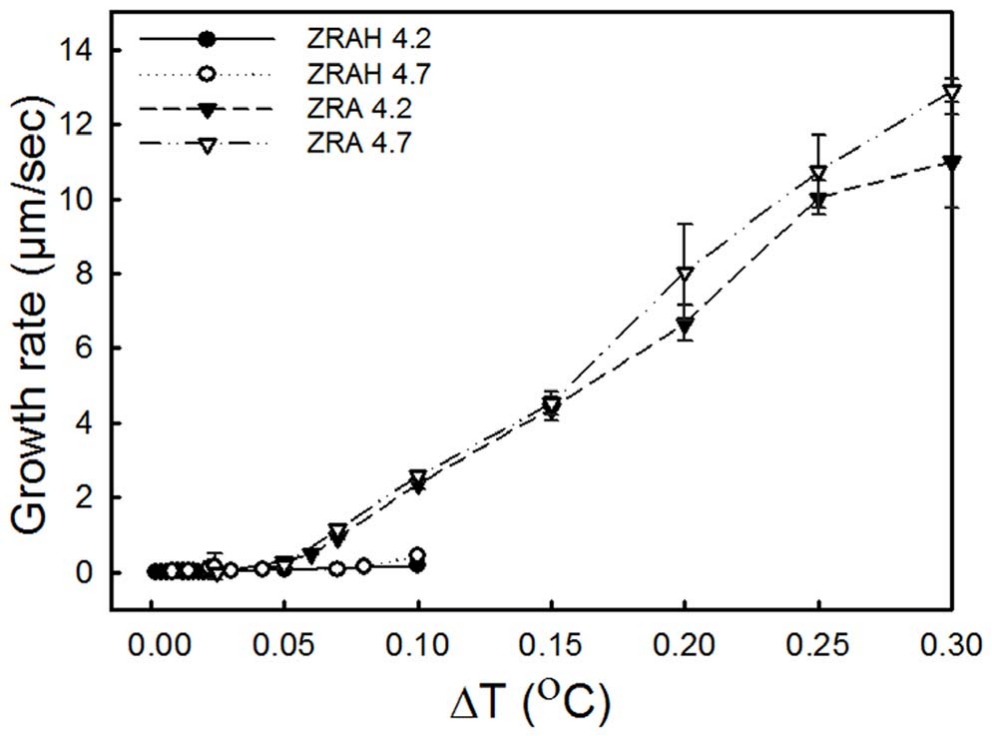
Comparison of the effects of ZRAH and ZRA on the ice growth rates along the *c*-axis. The X-axis represents the temperature below T_m_. The Y-axis represents the rate of growth of the radius of a seed ice crystal 20 µm in length. For ZRAH solutions, growth at ΔT >0.1°C could not be measured due to crystal orientation.

Crystals in ZRAH and ZRA solutions at pH 3.3 did not significantly affect the ice growth rate along the *a*-axis compared to buffer. However, at pH values of 4.2 and 4.7 the growth rate of the ice crystals was dramatically decreased (Fig. 2–3). Growth inhibition was significantly affected by the solution pH, with a higher activity observed in solutions with a higher pH. ZRA was significantly more active than ZRAH. Over a certain range of temperatures, the growth rate along the *a*-axis was less than 0.02 µm/s (Fig. 2b). This slow growth rate is 10 times slower than the rate previously used as the threshold for the freezing temperature in thermal hysteresis (TH) measurements, marking the boundary between no growth and growth conditions [26]. In such thermal hysteresis measurements, the melting temperature of a single ice crystal is determined, and then the crystal is cooled down in a slow rate until growth is detected, and the difference between the melting and freezing temperatures is determined as the TH activity of the sample. Supercooling temperatures at which the growth rates were below 0.2 µm/s were considered within the TH gap [26]. The accuracy of our equipment allows detection of slower growth rates and we defined the threshold for TH activity to be 0.02 µm/s. According to this definition, ZRA and ZRAH exhibited TH activity along the *a*-axis, as indicated by the arrow in Fig. 2b. Nevertheless, observation of an ice crystal in ZRAH at pH 4.2 over long periods of time (up to two hours) at temperatures within the semi-TH gap (0.060°C below T_m)_ revealed slow but steady growth at a rate of 0.01 µm/s along the *a*-axis. Notably, this growth rate was about 600 times slower than the growth rate observed in a buffer solution for the same supercooling level.

The growth of ice crystals along the *c*-axis in the presence of ZRA and ZRAH was measured. The relatively high growth velocities along the *a*-axis in the solutions at pH 3.3 resulted in flat shapes, which forced the crystals to float such that the *c*-axis was parallel to the view direction. This orientation limited our ability to measure the growth rate along the *c*-axis (Fig. 3). Note that in ZRAH, the growth velocity along the *c*-axis was lower than in ZRA under a supercooling of 0.05–0.1°C. This behavior may have resulted from latent heat release during the fast growth in the *a*-axis direction, which increased the temperature at the basal plane.

### IRI Activity

**Figure 4:**
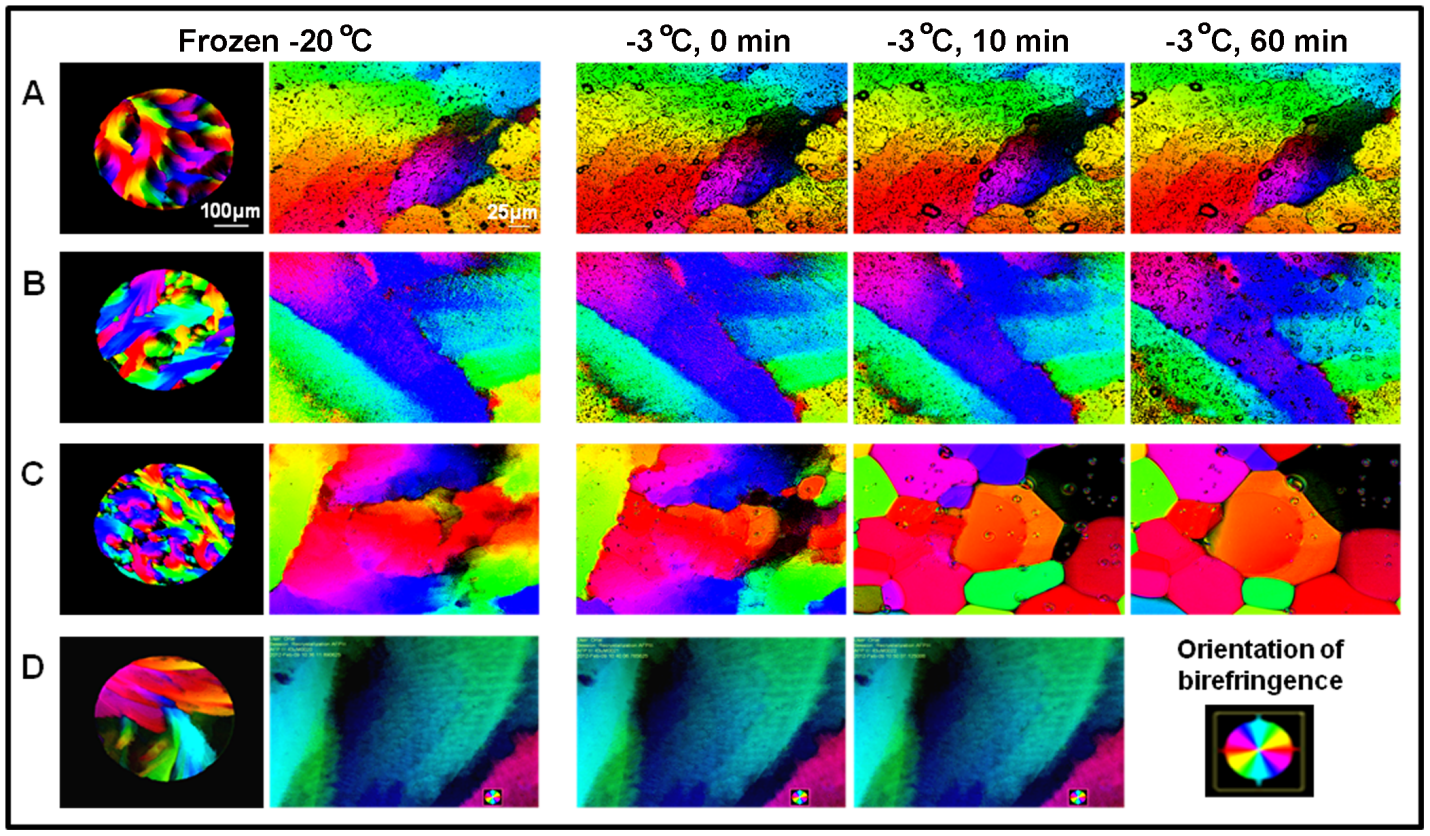
Ice recrystallization inhibition by ZRA and ZRAH. LC-PolScope images for (A) ZRA at pH 4.2, (B) ZRAH at pH 4.2, (C) 100 mM sodium acetate pH 4.2 (negative control), and (D) AFP III (40 µM solution). The colors represent the optical axis orientations of the ice which was determined by the Abrio birefringence detection system. The color map in the bottom right corner corresponds to the respective birefringence orientation. The images in the first and second columns show the initial frozen state at 4× and at 50× magnifications, respectively. Images in the last three columns were collected at an annealing temperature of –3°C, at times 0, 10, and 60 minutes. Note the grain boundaries that appeared during annealing only in the buffer sample. These boundaries shifts indicate recrystallization process.

ZRA and ZRAH were examined qualitatively for their IRI activities relative to the acetate buffer negative control or fish AFP type III positive control. The frozen samples were warmed to the annealing temperature (–3°C), and recrystallization appeared immediately in the acetate buffer sample (Fig. 4), but not in the ZRAH or ZRA samples. Delicate signs of change were apparent in the case of ZRA at pH 4.7, as observed in Fig. 4a. ZRAH efficiently prevented recrystallization of ice at all pH values studied. The ice observed during annealing did not change over a time course of 60 minutes, similar to the behavior observed in solutions containing the AFP type III. Ice recrystallization in the acetate buffer was clearly observed after this time period, as indicated by the pattern change in the images. Notably, the acetate buffer appeared to contain more ice crystals in the initial freezing state than in the ZRAH or AFP type III solutions (Fig. 4c, first column).

## Discussion

### TH Activity

The findings of this study showed that the inorganic synthetic materials ZRA and ZRAH exhibited certain traits that are characteristic of AFPs. A comparison of the growth of ice in a buffer solution or in the presence of ZRA or ZRAH indicated a hundred-fold reduction in the growth rates along the *a*-axis compared to the growth in buffer over the given temperature range. Nevertheless, whereas AFPs can completely halt ice growth over a range of temperatures, ZRA and ZRAH could not. The observation of continuous growth along the direction of the *c*-axis indicated that ZRA and ZRAH did not produce true TH activity, as is observed in the presence of antifreeze proteins. We note that although the hyperactive AFPs inhibit ice growth along the basal plane [29], [30], [31], [32], [33], moderate AFPs do not block basal plane growth; however, moderate AFPs clearly induce ice crystals to grow such that the prism plane is tilted, resulting in a bi-pyramidal shape that eliminates the presence of a basal plane altogether. Both Moderate and hyperactive AFPs completely halt crystal growth within the TH gap. These properties were not observed in the ZRA or ZRAH solutions in which ice crystals assumed columnar shapes with continuous basal growth. Still, over a defined temperature range, ice growth along the direction of the *a*-axis was reduced to less than 0.02 µm/s, which is 10 times below the rate that was previously considered to be “no growth” and, therefore, considered to be TH activity [26]. One can claim that ZRA and ZRAH have directionally-limited TH activity. Nevertheless, in our experimental resolution we were able to note that after a few hours, slow but continuous growth of the crystal to the *a* direction was observed also within this temperature range, indicating that the interactions between ZRA or ZRAH and ice are not as strong as the interactions of AFPs. The mechanism by which ZRA inhibits growth must, therefore, differ from the mechanism by which AFPs interact with ice, as noted by Deville et al. [19].

In addition to growth rate reduction, melting inhibition was observed. ZRAH at pH 4.7 produced a melting rate of 0.2 µm/s for ice at a temperature of 0.01°C above the T_m_, which is ten times slower than the melting rate of ice in an acetate buffer at the same temperature (2.1 µm/s). Such melting effects are characteristic of AFPs, as have been demonstrated by Celik et al. [34] and Bar-Dolev et al. [29]. Stabilization of the ice front against melting indicates a direct interaction between the molecules of the ZRA (and ZRAH) complexes and the receding ice front.

### IRI Activity

The presence of ZRAH or ZRA dramatically slowed or completely stopped ice recrystallization compared to the buffer. Similar IRI results were obtained for the AFP type III positive control. Although recrystallization was observed for only a relatively short period of time, the effects of the compounds on IRI were clear.

### Ice Shaping Activity

The ice shaping properties of ZRA and ZRAH, which led to hexagonal columnar growth patterns, along with the growth inhibition activities to the *a*-axis direction, suggested that these compounds were active on surfaces parallel to the ice prism planes or some other planes that were slightly tilted with respect to the prism planes. Moderate AFPs have been shown to interact with the prism planes of ice and surfaces slightly tilted with respect to these planes [35], [36], [37], [38]. In some cases (AFP type I), interactions with the pyramidal planes have been proposed [37], [38], and in others, multiple binding sites appear plausible (AFP III) [36], [39], [40]. In either case, no direct interactions between the moderate AFPs and the basal planes of ice are observed; however, the AFPs induced bipyramidal ice shapes with minimal or no exposed basal planes. Seed ice crystals grown in a solution containing moderate AFPs display initial growth along the *c*-axis. As growth along the *c*-axis progresses, the basal planes shrink until they are reduced and the crystal eventually acquires a bipyramidal shape and is protected from growth in any direction. Despite the fact that these AFPs do not directly interact with the basal planes [29], [41], the crystals are protected from all directions, and growth is arrested throughout the TH gap. Ice assumes hexagonal columnar shapes in the presence of ZRA or ZRAH solutions. The basal planes are, therefore, exposed and not well protected, leading to continuous growth along the *c*-axis. The differences between the growth patterns of ice in the presence of AFPs, ZRA, or ZRAH can be explained according to differences in the adsorption planes or sites on the ice surfaces. Whereas AFPs may bind to more than one plane [40], ZRA and ZRAH may not. One possible mechanism for AFP binding relies on irreversible binding [23], [34], [42]. The binding of ZRA and ZRAH may well be reversible, which can explain the slow growth along the *a*-direction observed after hours of incubation.

Hexagonal column-shaped crystals and continuous slow growth were previously observed in the presence of mutated AFPs [25], [26] and antifreeze glycoprotein (AFGP) analogs [27]. In the latter, the slow growth was explained by a reduction in the adsorption constants of the protein analogues to ice, an explanation that may be valid for ZRA and ZRAH. Our data, nevertheless, does not rule out the possibility that the observed activity stemmed from the effects of the compounds on the water structure. ZRA and ZRAH may act at the quasi-liquid layer and interact with the water molecules there. This mechanism was proposed previously as underlying the interactions in carbohydrate or glycopeptide solutions, which displayed IRI without influencing the dynamics of ice shaping [43]. Recent studies have showed that AFGPs affect water molecules in the far hydration shell and, therefore, support this possible mechanism [44].

### Differences between the ZRA and ZRAH Activities

While little is known about the actual structure of ZRA in an aqueous solution [45], it is known that ZRA tends to polymerize upon aging, heating, or a pH increase [45], [46]. The monomeric units of ZRA prior to polymerization may consist of four zirconium atoms arranged in a tetrameric form, and these tetramers can arrange themselves in organized rod-like structures linked by double hydroxyl bridges [46]. Indeed, we noted that some of our solutions, mainly those at high pH, gelatinized after a few hours or days at room temperature. Studies of zircolyl chloride solutions have shown that oligomerization of a tetramer and an octamer zirconyl species occurs by heating, aging, or reduction of solution acidity [47], [48]. Similar polymerization processes may occur in solutions obtained either by dissolving ZRAH in an acetate buffer or by dissolving ZRA in an acetic acid and water solution. The active species in both ZRA and ZRAH may, therefore, be the same or very similar oligomeric forms. The lower activity of ZRAH relative to ZRA stems from the lower concentration of the active species in solution. Deville et al. claimed that ZRAH solutions displayed no ice shaping activities [19]. We showed that ZRAH produced meaningful ice recognition, as indicated by its IRI and ice growth inhibition activity.

### pH Dependence of Ice Growth Retardation and IRI

The pH dependence of ice growth inhibition by ZRA and ZRAH solutions may be due to differences in the complex structures, stoichiometry, or oligomerization in solution, as indicated above. Solutions containing a Zr:acetate ratio of 1∶2 (at concentrations of 0.01 or 0.02 M) were found to exhibit a dramatic change in the main complex species between pH 3 and 4 [49]. The authors attributed this change to a complex transition from one acetate group per Zr atom to two acetate groups per Zr. Studies of Zr in aqueous solutions revealed that the dominant species at low pH is the tetrameric form [Zr_4_(OH)_8_(H_2_O)_16_]^8+^ [50]. Later on, it has been demonstrated that an octameric species of Zr is in equilibrium with the tetramer, and that the octamer is predominant in solutions of lower acidity [47]. Other studies revealed that pH increase as well as aging of Zr(IV) aqueous solutions result in larger oligomeric species [51]. A different study described that pH increase or reflux treatment of acidic zirconyl solutions resulted in oligomerization of the tetrameric units into two-dimensional sheets that later on formed gels and percipitate [48]. We associate the correlation between the increase in ice growth inhibition activity and the decrease in acidity of the solutions to an enhancement of the oligomerization at higher pH levels, thereby increasing the amount of the active species in solution. Active oligomeric zirconium acetate may either be in the form of chains or in the form of sheets. The slight reduction in the growth inhibition activity of ZRA at pH 4.7 relative to 4.2 may have been due to over-polymerization. Partial oligomerization may be important for ice recognition because more ZRA units per molecule increase the number of interactions with ice; however, over-polymerization can also disturb the ice recognition activity due to slow diffusion of the heavy molecules or to an increase in the presence of non-active species in solution. An extensive spectroscopic study of Zr(IV) complex formation in acetic acid or aqueous solutions showed that the stoichiometry of an acetate complex of Zr(IV) is independent of the Zr/acetic acid ratio, the Zr concentration, or the solution pH [52]. This study supports the notion that the main difference between the samples prepared at different pH values was the oligomerization state.

Deville et al. observed hexagonal shapes in the ice crystals at pH 4 but not at pH 4.5 or 3.3. The author suggested that the decrease in ice shaping activity at these pH values arose from surface charge repulsion between the ice and ZRA [18]. Contrasting with these findings, we observed ice recognition activity by ZRA at both pH 3.3 and 4.7. The activity of ZRAH was higher at pH 4.7 than at pH 4.2, precluding the possibility of charge repulsion at this pH range. The differences between our findings and those of Deville et al. may stem from the different procedures of sample preparation as well as differences in the assays. Notably, we found that ZRAH at a pH as low as 3.3 was sufficient for IRI but not for growth inhibition or ice shaping. Excellent IRI, along with a low TH activity, have been observed in solutions of the AFP derived from ryegrass [53].

### Ice Recognition by ZRA and ZRAH

**Figure 5:**
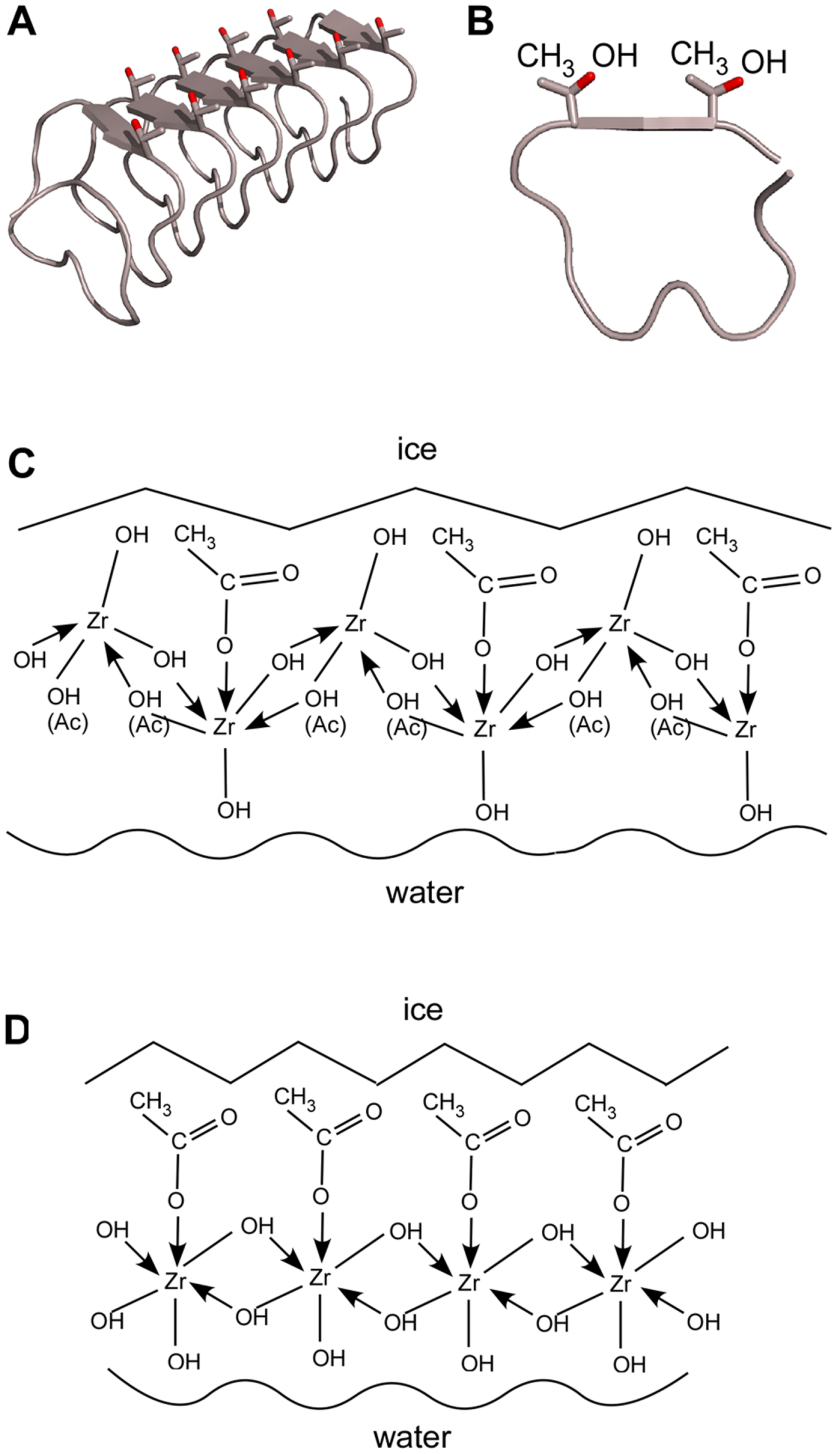
Proposed mechanism for ice recognition by ZRA and ZRAH. A–B. Representation of the Structure of AFP from mealworm beetle (*Tenebrio molitor*) (*Tm*AFP), showing the backbone as a ribbon and the threonine residues forming the ice-binding face in a ball-and-stick model. Oxygen atoms are presented in red and carbon atoms in gray. (A) Side view of the protein. (B) Cross-section of the structure. (C–D) Models suggested for the interactions of ZRA and ZRAH with ice. See text for details.

The functional groups that may participate in ice recognition by the hydroxyl-bridged ZRA oligomer are the acetate and hydroxyl moieties. The acetate group is linked to the Zr(IV) ion through an oxygen atom, leaving a methyl group and another oxygen or hydroxyl group exposed to the solvent. This arrangement is similar to the side chain of the threonine amino acid, which plays a significant role in the ice binding faces of many AFPs [30], [31], [54]. The acetate groups in a ZRA polymer are thought to be exposed on one side of the chain, along with hydroxyl groups, whereas the other side of the chain exposes only hydroxyl groups. The ZRA polymer is thought to be neutral in charge [55]. The part of the polymer that presents the acetate groups may, to a certain extent, mimic the ice binding sites of several ice-binding proteins. The crystal structures [30], [31], [54] as well as mutagenesis studies [56], [57] of several AFPs have revealed that the ice binding faces of these proteins consist of an array of threonine residues with side chains exposed on the outer surface of the protein and arranged in such a way as to match certain planes of the ice crystals nearly perfectly. Furthermore, it has been shown that the hydrophobic part of the ice-binding faces is important for ice recognition while the more hydrophilic portions of the proteins are solvent-exposed [56], [58], [59]. A similar ice-binding mechanism can be envisioned for ZRA, as illustrated in Fig. 5. Here, a much less perfect match is achieved, offering a possible explanation for why ZRA only incompletely arrests ice growth.

On this basis, we propose two structural arrangements by which ZRA can bind to an ice surface (Fig. 5). In the first model, a methyl group from the acetate and an adjacent hydroxyl group act as the ice recognition unit. In this model, the acetate groups of ZRA are oriented in multiple directions, which is possible for short-chain oligomers or small two dimensional clusters. In the second model, adjacent acetate groups act as the ice recognition site. The interactions between ZRA and ice are then mainly governed by van der Waals forces involving the methyl group, similar to the interactions observed in a heavily mutated AFP in which most of the threonine residues on the ice binding face were replaced with valine, thereby exposing only methyl groups to the solvent. This mutant showed a low TH activity but was very efficient in ice shaping and ice growth inhibition [25]. Although it is unlikely that ZRA interacts with ice via an anchored clathrate mechanism [54], ZRA may interact with ice through a mechanism similar to that employed by AFP mutants. Deville et al. recently stated that it is more likely that ZRA interacts with ice through its hydrophilic side through a mechanism that differs from that observed among AFPs; however, the authors did not rule out the possibility of interactions with the hydrophobic side [19].

Note that although the models presented here consist of chains and assume that preliminary polymerization steps have occurred, the interactions are valid for other ZRA structures that may be present in solution, such as a tetramer in which four zirconium atoms are bound by hydroxo [46] or oxo [52] bridges with a ratio of one [52] or two [49] acetate groups per zirconium atom.

The effects of the ZRA compound on the ice crystal growth or melting may be advantageous for cryopreservation. The efficient inhibition of ice recrystallization and the induction of smooth growth patterns without spontaneous “burst” growth, as are observed in the presence of AFPs, could prevent fatal damage to cells, tissues, and organs. For example, the addition of AFP type I [60], [61] or type III [61] was accompanied by extensive growth of ice around the cell during cooling, which damaged the cells, presumably due to the spicular growth pattern.

### Conclusions

This work examined the extent to which ice growth could be controlled in the presence of ZRA and ZRAH. These compounds were shown to share many of the properties of natural AFPs, including IRI activities, ice-shaping activities, and a reduction in the ice growth and melting rates. The structures of ZRA complexes and polymers suggested that ZRA interacts directly at the ice:water interface, although further studies are needed to elucidate the mechanism of action underlying the ZRA effects. While ZRA do not have high TH activity, it presents several practical advantages over AFPs, including a low production cost, good stability, and abundance in nature. ZRA is an excellent candidate agent that warrants further study for possible use in applications in which ice growth control remains a challenge.

## Supporting Information

Figure S1 **Ice crystal growth as truncated pyramidal shapes in a solution containing ZRAH at pH 4.7.** The images were taken at a temperature 0.040°C below the melting point.
(TIF)

Figure S2 **Hopper shapes induced by ZRAH.** Ice crystal grown at a high supercooling rate in the presence of ZRAH at pH 4.7 developed hopper shape with increased basal planes that appeared to be hollow. The sequence of images was taken during the growth course. Frame A was collected before the temperature had been reduced, at the melting point. The other three images were taken during temperature decline. The actual temperature below the melting point and the time lapse is presented. The arrows in A denote the crystal orientation in the sample.
(TIF)
