# Supplementary figures and images for: Inhibition of Ice Growth and Recrystallization by Zirconium Acetate and Zirconium Acetate Hydroxide
Source: PLoS One. 2013 Mar 21;8(3):e59540. doi: 10.1371/journal.pone.0059540 (PMC3605400; doi:10.1371/journal.pone.0059540)

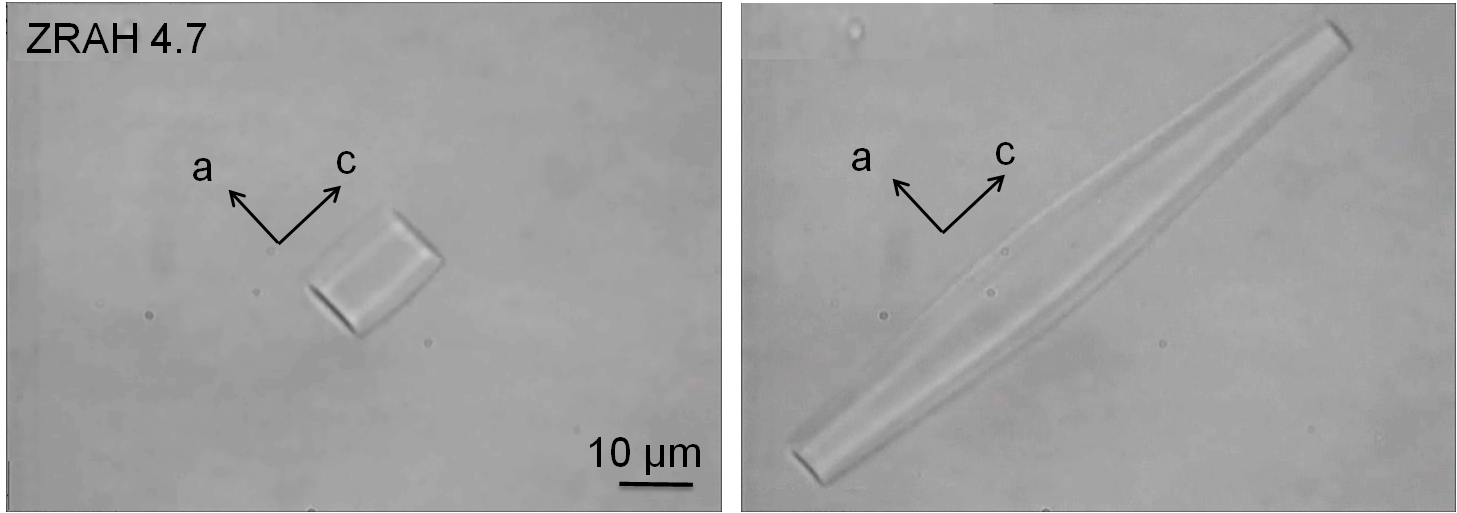

Supplement: Figure S1 — Ice crystal growth as truncated pyramidal shapes in a solution containing ZRAH at pH 4.7. The images were taken at a temperature 0.040°C below the melting point. (TIF) [file pone.0059540.s001.tif]

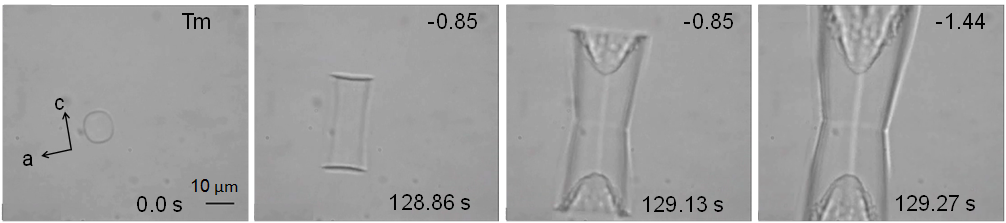

Supplement: Figure S2 — Hopper shapes induced by ZRAH. Ice crystal grown at a high supercooling rate in the presence of ZRAH at pH 4.7 developed hopper shape with increased basal planes that appeared to be hollow. The sequence of images was taken during the growth course. Frame A was collected before the temperature had been reduced, at the melting point. The other three images were taken during temperature decline. The actual temperature below the melting point and the time lapse is presented. The arrows in A denote the crystal orientation in the sample. (TIF) [file pone.0059540.s002.tif]
